# Neuroprotective Effects of Sodium Butyrate and Monomethyl Fumarate Treatment through GPR109A Modulation and Intestinal Barrier Restoration on PD Mice

**DOI:** 10.3390/nu14194163

**Published:** 2022-10-07

**Authors:** Rui-Chen Xu, Wen-Teng Miao, Jing-Yi Xu, Wen-Xin Xu, Ming-Ran Liu, Song-Tao Ding, Yu-Xin Jian, Yi-Han Lei, Ning Yan, Han-Deng Liu

**Affiliations:** 1 Laboratory of Tissue and Cell Biology, Experimental Teaching Center, Chongqing Medical University, Chongqing 400016, China; 2 College of First Clinical, Chongqing Medical University, Chongqing 400016, China; 3 College of Pediatrics, Chongqing Medical University, Chongqing 400016, China; 4 Department of Neurology, University-Town Hospital of Chongqing Medical University, Chongqing 400016, China; 5 Molecular Medicine and Cancer Research Center, Department of Cell Biology and Genetics, Chongqing Medical University, Chongqing 400016, China

**Keywords:** Parkinson’s disease, intestinal barrier, sodium butyrate, GPR109A receptor, monomethyl fumarate

## Abstract

Research has connected Parkinson’s disease (PD) with impaired intestinal barrier. The activation of G-protein-coupled receptor 109A (GPR109A) protects the intestinal barrier by inhibiting the NF-κB signaling pathway. Sodium butyrate (NaB), which is a GPR109A ligand, may have anti-PD effects. The current study’s objective is to demonstrate that NaB or monomethyl fumarate (MMF, an agonist of the GPR109A) can treat PD mice induced by 1-methyl-4-phenyl-1,2,3,6-tetrahydropyridine (MPTP) via repairing the intestinal barrier. Male C57BL/6J mice were divided into four groups randomly: control, MPTP + vehicle, MPTP + NaB, and MPTP + MMF. Modeling mice received MPTP (20 mg/kg/day, i.p.) for a week, while control mice received sterile PBS. Then, four groups each received two weeks of sterile PBS (10 mL/kg/day, i.g.), sterile PBS (10 mL/kg/day, i.g.), NaB (600 mg/kg/day, i.g.), or MMF (100 mg/kg/day, i.g.). We assessed the expression of tight junction (TJ) proteins (occludin and claudin-1), GPR109A, and p65 in the colon, performed microscopic examination via HE staining, quantified markers of intestinal permeability and proinflammatory cytokines in serum, and evaluated motor symptoms and pathological changes in the substantia nigra (SN) or striatum. According to our results, MPTP-induced defected motor function, decreased dopamine and 5-hydroxytryptamine levels in the striatum, decreased tyrosine hydroxylase-positive neurons and increased activated microglia in the SN, and systemic inflammation were ameliorated by NaB or MMF treatment. Additionally, the ruined intestinal barrier was also rebuilt and NF-κB was suppressed after the treatment, with higher levels of TJ proteins, GPR109A, and decreased intestinal permeability. These results show that NaB or MMF can remedy motor symptoms and pathological alterations in PD mice by restoring the intestinal barrier with activated GPR109A. We demonstrate the potential for repairing the compromised intestinal barrier and activating GPR109A as promising treatments for PD.

## 1. Introduction

Parkinson’s disease (PD) is one of the most prevalent neurodegenerative illnesses in the world with complex and progressive pathological processes [1]. It was first described by Dr. Parkinson approximately two centuries ago; however, it is frustrating that the exact etiology of PD is still uncertain at present. The causes of PD are multifactorial, and both hereditary and environmental factors may contribute to its development. Research suggests that oxidative stress and neuroinflammation presumably account for major pathogenesis [1]. The mitochondria, which are cellular organelles important for energy production, have long been supposed to be altered in PD via oxidative stress [2]. Doric and Nakamura have proposed a new model of PD that involves dysfunctional mitochondria [3]. The appearance of Lewy bodies and excessive death of dopaminergic neurons in the substantia nigra pars compacta (SNpc) are the two most distinguishing characteristics of its histopathology [4]. Resulting from the destruction of neurons, motor symptoms such as rest tremor, bradykinesia, rigidity, and gait impairment are the hallmark clinical signs of PD. Additionally, hyposmia, cognitive decline, sleep issues, and other nonmotor symptoms (NMS) are also common among PD patients. Gastrointestinal conditions such as constipation, nausea, vomiting, dysphagia, and delayed gastric emptying have drawn great attention recently [5,6,7]. The fact that these gastrointestinal symptoms appear before typical motor symptoms raises the possibility that the gastrointestinal system and PD are related. Currently, mainstream treatments for PD involve drugs such as levodopa, catechol-o-methyl transferase (COMT) inhibitors, monoamine oxidase B (MAO-B) inhibitors, anticholinergics, and functional neurosurgery techniques such as deep brain stimulation [8]. However, it is a pity that these strategies cannot reverse the neurodegenerative process in the brain. In other words, current treatments for PD are ineffective. More in-depth explanations of the mechanisms of PD are necessary for developing effective therapy techniques. Given the studies that PD may be strongly linked to a sick gut and may originate from the gut before affecting the brain, approaches that target gastrointestinal abnormalities in PD hold promise.

**Figure 1:**
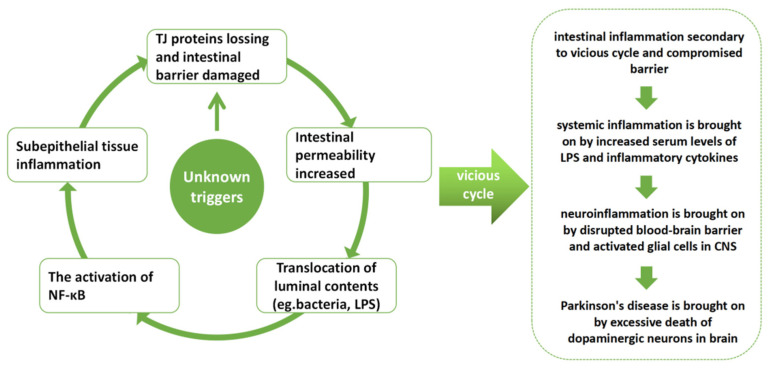
According to our hypothesis, the deficiency of tight junction proteins and enhanced gut barrier permeability, which will allow for the translocation of bacteria and other luminal content, local activation of the NF-κB signaling pathway, and inflammation of the intestinal wall tissue, are all key processes for the vicious cycle secondary to the damaged intestinal barrier. Eventually, inflammation creates a vicious loop by aggravating the damage to the intestinal barrier.

The “gut-brain axis” theory, which suggests that intestinal flora or other variables in the gut are intimately related to PD progression, has attracted a lot of attention recently [9]. A 2003 paper by Braak and his colleagues reported the appearance of intestinal Lewy bodies before the formation of Lewy bodies in SNpc, supporting the theory that PD may have its root in the enteric nervous system [10]. A dysfunctional intestinal barrier is one of many elements that can have an impact on brain function. As the initial line of protection for the body, the intact gut barrier is indispensable for maintaining homeostasis by shielding host tissues from environmental toxins and pathogens. If this defense is ruined, a sequence of pathological processes will be initiated. It is widely accepted that inflammatory bowel diseases (IBDs) and compromised intestinal barriers are related [11,12,13]. A large number of predisposing genes such as LRRK2, GALC, GPR65, and FCGR2A are shared between IBDs and PD [14]. PD is more common in people with IBD than in the general population, pointing to the potential connection between PD and compromised intestinal barrier. Furthermore, several investigations have shown that PD patients have a deficiency in the intestinal barrier. Occludin and claudin are crucial tight junction (TJ) proteins that help build the epithelial barrier and were shown to be expressed less in the gut of PD patients [15]. Sven and his colleagues found that the blood and feces of PD patients had increased concentrations of zonulin, another type of significant TJ protein [16]. Moreover, the damaged intestinal barrier was also discovered in animal models for PD [17]. As a result, the dysfunction of the intestinal barrier caused by several factors is prone to trigger pathological progress such as the translocation of bacteria, the imbalance of gut flora, and finally cause microinflammation in interepithelial, subepithelial, and distant areas [18]. In our hypothesis, the vicious cycle resulting from the pathogenic process described above may trigger or exacerbate the development of PD, caused by leaky gut, the translocation of luminal antigens and toxicants are capable of initiating local as well as systemic inflammation, and the permeability of the blood-brain barrier will also be increased secondarily to increased pro-inflammatory cytokines in the blood afterward. Enteric lesions may affect the brain through the pathological pathway described above. We have drawn a picture to illustrate our hypothesis (Figure 1).

We have reviewed the potential roles of microbial metabolites in PD [19], as one of the major components in microbial metabolites, the level of butyrate was thought to have an impact on the development and progression of PD. Some documents have reported the therapeutic effects of sodium butyrate (NaB) in the PD model despite that the precise mechanism of beneficial effects is unknown [20,21]. In IBD model mice, activating the G-protein-coupled receptor 109A (GPR109A) receptor may have protective effects by suppressing the NF-κB signaling pathway and preserving the intestinal barrier [22]. According to a study by Fu and colleagues, β-Hydroxybutyric acid (BHBA) can shield dopaminergic neurons against neuroinflammation by modifying GPR109A [23]. Furthermore, NaB is a type of typical GPR109A ligand [24]. Feng has reported that NaB attenuates diarrhea while promoting the expression of TJ proteins in weaned piglets [25]. These findings imply that the GPR109A’s anti-inflammatory effects on the NF-κB pathway and repair of the damaged intestinal barrier may be necessary for NaB’s possible protective effect in PD. However, there are no published lectures demonstrating that GPR109A activation might be the underlying mechanism of NaB in PD. As we have illustrated above, provided the destructed intestinal barrier in PD, strategies that focus on the gut are promising.

The aim of the current research is to demonstrate how sodium butyrate prevents PD by preserving and repairing the damaged intestinal barrier. Additionally, in an effort to research the activation of the GPR109A receptor’s independent positive effect, in place of NaB, we added a treatment group that used monomethyl fumarate (MMF), a type of GPR109A agonist [26]. Results about GPR109A and NF-κB activation revealed the significant role of GPR109A in PD, these findings provide preliminary support for targeting the damaged intestinal barrier and activating the GRP109A receptor as novel PD therapies. 

## 2. Materials and Methods

### 2.1. Animals and Drugs

Male C57BL/6J mice aged seven weeks and weighing 20–22 g were purchased from Chongqing Byrness Weil biotech Ltd. Mice were acclimatized in a sterile environment (temperature 22 ± 2 °C, humidity 50–60%, 12 h light and 12 h dark alternately) for a week. Mice were given adequate food and water and were weighed every day. Drugs (MPTP, NaB, and MMF) were diluted to appropriate concentrations with PBS. All experimental procedures were approved by the Animal Ethics Committee of Chongqing Medical University.

### 2.2. Groups and Experimental Procedure

**Figure 2:**
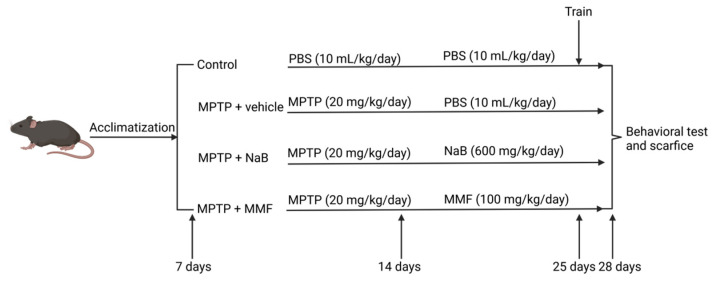
The treatment flow chart for animals.

The groups and experimental procedures are shown in Figure 2. Four groups of mice were randomly assigned to each other: the control group, the MPTP + vehicle group, the MPTP + NaB group, and the MPTP + MMF group. In the first week, mice in all groups other than the control group received an intraperitoneal injection of MPTP (20 mg/kg/day) [27], while the same quantity of sterile PBS was given to mice in the control group. In the second and third weeks, the MPTP + NaB group and the MPTP + MMF group respectively received gavage treatments of NaB (600 mg/kg/day) and MMF (100 mg/kg/day) [28,29], and PBS (10 mL/kg/day) was gavaged to both the control group and the MPTP + vehicle group. 

### 2.3. Behavioral Tests

Three different behavioral tests, including the rotarod test, traction test, and pole test were carried out to examine the motor function of mice [30,31,32]. Mice were trained once each day from the 12th to the 14th day. On the 15th day, mice were assessed three times, with an hour separating each session.

#### 2.3.1. Rotarod Test

We kept the rod’s rotation at 30 RPMs for up to the 180 sec. Each mouse’s latency, or the moment it slipped off the rotarod, was automatically recorded [30]. Three times, with an hour in between, were performed on each mouse. Mice with results below 3 sec were examined again. The final outcome is an average of three times latency.

#### 2.3.2. Traction Test

Mice were suspended from the wire by their forepaws [31]. The evaluation criteria were as follows: The mice hooked on the wire with four limbs achieved four points. Mice with three limbs on the wire achieved three points, and two points were awarded to the mice who had two forepaws on the wire. One point was awarded to the mice who only had one paw on the wire. Each mouse underwent the exam three times. Each mouse’s overall score is represented by the average.

#### 2.3.3. Pole Test

With 2 cm spherical protuberance on the top, a 50 cm wooden pole was wrapped around a circle of gauze to prevent the mice from slipping, and it was placed on the ground [32]. The mice were timed as they descended the pole while lying head-down with four paws on top. The mice’s descent of the pole was timed while they were positioned head-down with four paws on top. Timing started when the researcher let go of the mouse’s tail and ended when all four of the mouse’s paws touched the ground. Each mouse repeated the test three times, and the final result was the average of the three times.

### 2.4. Sacrifice and Sample Processing

To relieve the pain, isoflurane was used to sacrifice the mice. Following anesthesia, mice of each group for immunofluorescence were given cardiac perfusion using 30 mL pre-cooled sterile saline and 30 mL pre-cooled paraformaldehyde (PFA) per mouse. After cardiac perfusion, distal colons and brains were taken and immersed in PFA. Simultaneously, other mice were given cardiac perfusion of pre-cooled sterile saline 30 ml per mouse. Fresh striatum and distal colon tissues were taken and kept at −80 °C after the perfusion. The upper serum was separated from the blood by centrifuging at 2000× *g* at 4 °C for 20 min. The upper serum was then stored at −80 °C.

### 2.5. Hematoxylin and Eosin Staining

Hematoxylin and eosin staining was used to examine the integrity and inflammatory state of the intestinal barrier. The colon tissue was taken out, fixed in PFA at a concentration of 4% for 12 h, and then refrigerated at 4 °C. After becoming translucent, the tissues were dried off, and paraffin wax was added to them. Hematoxylin and eosin was used to stain the 4 μm thick slices of paraffin blocks that were cut. According to Hollingshead [33], the assessment of colonic pathological alteration and graded inflammation was made using a coincident standard. 

### 2.6. Enzyme-Linked Immunosorbent Assay

Diamine oxidase (DAO) and D-lactate (D-LA) are two markers of intestinal permeability. ELISA kits (CSB-E10090m, CUSABIO, Wuhan, China; JLC084, JKBIO, Shanghai, China) were used to assess the serum level of DAO and D-LA. The entire procedure was carried out in accordance with the manufacturer’s instructions. According to the manufacturer’s instructions, IL-6 ELISA Kit (EK0411, BOSTER, Wuhan, China) and TNF-ELISA Kit (EK0527, BOSTER, Wuhan, China) were used to determine the serum concentrations of the inflammatory factor interleukin-6 (IL-6) and tumor necrosis factor-α (TNF-α). At 450 nm, the OD value was measured.

### 2.7. Immunofluorescence Staining

The dissected brain tissues were immediately immersed in 4% PFA for 24 h and embedded in paraffin after a sequence of dehydration processing. The dewaxing processes were then carried out after the paraffin blocks were divided into 4 μm thick slides. The brain sections were then rinsed three times in PBS and placed in 0.01 M sodium citrate buffer (pH 6.0) for antigen retrieval. The sections were treated with a primary antibody overnight at 4 °C after being blocked with 10% Bovine Serum Albumin (BSA) (ST023, BEYOTIME, Shanghai, China) for an hour. Primary antibody: anti-tyrosine hydroxylase (TH, 1:200, Abcam, Cambridge, UK) and anti-ionized calcium-binding adapter molecule 1 (IBA-1, 1:100, CST, USA). After half an hour of rewarming, appropriate secondary antibody FITC-labeled goat anti-mouse IgG (1:500, Thermo Fisher, Waltham, MA, USA) was used to detect the primary antibody for 1 h, which was observed under the fluorescence microscope (Nikon eclipse 80 i; Nikon, Tokyo, Japan). The results of each group were analyzed by Image J software, version, 1.53 (NIH, Bethesda, MD, USA).

### 2.8. Measurement of Neurotransmitters

The content of striatal dopamine (DA), 5-hydroxytryptamine (5-HT), dihydroxy-phenylacetic acid (DOPAC, the metabolite of DA), and 5-hydroxyindoleacetic (5-HIAA, the metabolite of 5-HT), were detected by high-performance liquid chromatography (HPLC) with fluorescence detector and separation system and Atlantis T3 column. The mobile phase was filtered sodium citrate buffer (adjusted to pH 5 with hydrochloric acid). Standard samples of DA (H8502, Merck, Rahway, NJ, USA), 5-HT (A1824, Merck, Rahway, NJ, USA), DOPAC (850217, Merck, USA), and 5-HIAA (H8876, Merck, USA) dissolving in perchloric acid were diluted by gradient concentration and stored at −80 °C before the experiment. Meanwhile, frozen striatum samples were thawed and centrifuged at 12,000× *g* for 10 min. The supernatants were collected and stored in the ice bath. During the experiment, samples were filtered through 0.22 μm nylon filters before injecting them into the pump. The standard curves are obtained by detecting gradient concentrations of respective standard samples.

### 2.9. Western Blot

Colon tissues from the animals were removed and kept in the freezer at −80°C. Radioimmunoprecipitation assay lysis buffer (RIPA) (P0013B, BEYOTIME, Shanghai, China), Phenylmethanesulfonyl fluoride (A100754, Sangon Biotech, Shanghai, China), and phosphorylated protein inhibitors (P1050, BEYOTIME, Shanghai, China) were utilized to lyse the colon tissue. After centrifuging the lysate at 13,000× *g* for 10 min at 4 °C, the protein supernatant was collected, and the concentration of samples was assessed by BCA Protein Assay Kit (C503051-0500, Sangon Biotech, Shanghai, China). Then, we denatured protein with a metal bath and used sodium dodecyl sulfate-polyacrylamide gel electrophoresis (SDS-PAGE) (C631100-0200, Sangon Biotech, Shanghai, China) to separate samples. After being moved to the polyvinylidene difluoride membranes (IPVH00010, Millipore, Burlington, MA, USA), the sample was blocked overnight at 4 °C with 5% BSA. (ST023, BEYOTIME, China). Primary antibodies were incubated on the membranes for two hours at room temperature in a shaker. Primary antibodies: rabbit anti-GAPDH antibody (1:1000, 10494-1-AP, ProteinTech, Wuhan, China), rabbit anti-occludin antibody (1:1000, 27260-1-AP, ProteinTech, Wuhan, China), rabbit anti-claudin-1 antibody (1:1000, 13050-1-AP, ProteinTech, Wuhan, China), rabbit anti-GPR109A antibody (1:1000, PA5-90579, Thermo Fisher, Waltham, MA, USA), rabbit anti-NF-kB P65 antibody (1:1000, 4764T, Cell signaling technology, Shanghai, China). Then, the membranes were treated with the secondary antibody (1:1000, 7074S, Cell signaling technology, Shanghai, China) for an hour at ambient temperature. 

Finally, the EasyBlot ECL kit (D601039-0050, Sangon Biotech, Shanghai, China) was used to identify protein bands, and a Gel Image System (NIH, USA) was used to capture the images. The final results were analyzed using Image J software, version, 1.53 (NIH, USA). GAPDH was used as an internal control.

### 2.10. Statistical Analysis

The results were presented as mean ± SEM after statistical analysis was completed using Prism V.9 (La Jolla, CA, USA) and SPSS (SPSS, version, 26, Chicago, IL, USA) software. Multiple comparisons were made using one-way analysis of variance (ANOVA), and additional comparisons were made using either the Bonferroni test or the Tamhane T2 test. Statistics were considered significant for *p* values under 0.05.

## 3. Results

### 3.1. NaB and MMF Alleviated Behavioral Deficits of the MPTP-Induced PD Mice

**Figure 3:**
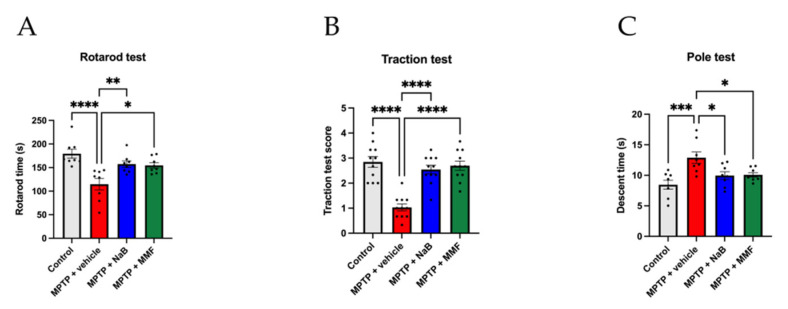
NaB and MMF alleviate motor dysfunctions of the MPTP-induced PD mice. (**A**) Rotarod test. (**B**) Traction test. (**C**) Pole test. (n = 8–11). Data are presented as mean ± SEM. * *p* < 0.05, ** *p* < 0.01, *** *p* < 0.001, **** *p* < 0.0001 versus the MPTP + vehicle group.

Bradykinesia, tremor, and impaired coordination are typical motor symptoms of PD. In three behavioral trials, the MPTP-induced mice group clearly underperformed the control group, showing motor impairments in PD. In the rotarod test, the MPTP + vehicle group experienced a decrease in latency in comparison to the control group (*p* < 0.0001 vs. control) (Figure 3A). Both the MPTP + NaB and MPTP + MMF groups significantly outperformed the MPTP + vehicle group (*p* < 0.01 and *p* < 0.05 vs. MPTP + vehicle, respectively) (Figure 3A), suggesting that NaB and MMF may alleviate impaired coordination in PD mice. In the traction test, the MPTP group scored less than the control group (*p* < 0.0001 vs. control) (Figure 3B). Comparatively, the MPTP + NaB group and MPTP + MMF group showed better motor performance than the MPTP group (*p* < 0.0001 and *p* < 0.0001 vs. MPTP + vehicle, respectively) (Figure 3B), indicating that NaB and MMF may have a therapeutic effect on tremor in PD mice. Thirdly, the MPTP + vehicle group showed a longer descent time compared to the control group (*p* < 0.001 vs. MPTP + vehicle) (Figure 3C). On the contrary, mice treated with NaB and MMF had shorter descent times (*p* < 0.05 and *p* < 0.05 vs. MPTP + vehicle, respectively) (Figure 3C), suggesting that these drugs may help bradykinesia. However, there was no significant difference between the MPTP + NaB group and the MPTP + MMF group in all tests. These results indicated similar therapeutic effects on motor dysfunction from NaB and MMF.

### 3.2. NaB and MMF Treatment Alleviated the Reduction of Brain Neurotransmitters in MPTP-Induced PD Mice

**Figure 4:**
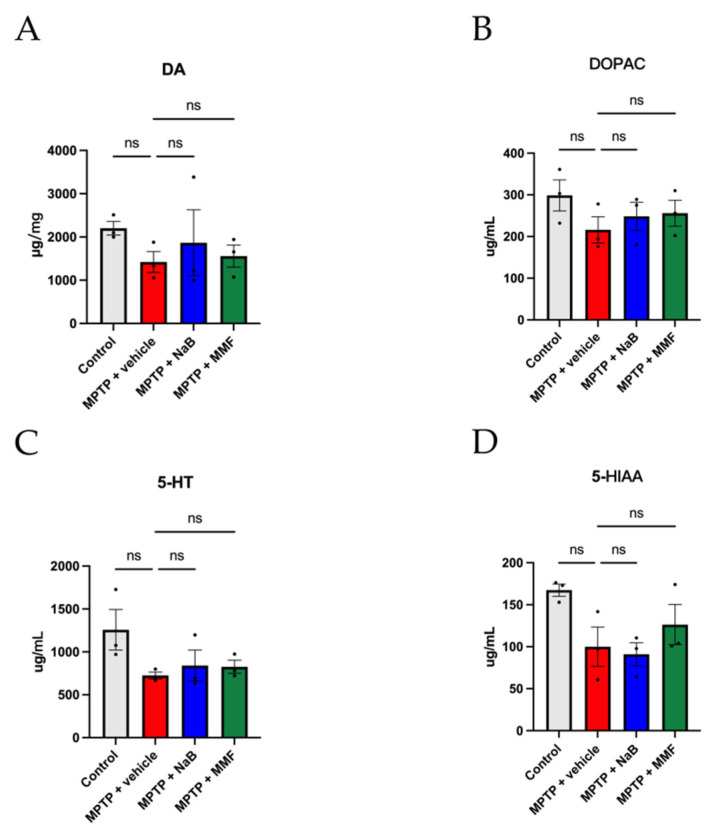
NaB and MMF treatment alleviates the reduction of brain neurotransmitters in MPTP-induced PD mice. (**A**) When compared to the MPTP + vehicle group, both the MPTP + NaB group and the MPTP + MMF group exhibit greater levels of DA in the striatum. (**B**) NaB and MMF boost the expression of DOPAC in MPTP-induced PD mice, as shown by the presence of DOPAC in the striatum. (**C**) NaB and MMF effectively corrected the 5-HT decrease in MPTP mice, as shown by the 5-HT content in the striatum. (**D**) The presence of 5-HIAA in the striatum indicates that NaB and MMF boosted the expression of 5-HIAA in PD animals. The content of DA, 5-HT, DOPAC, and 5-HIAA was quantified by HPLC. (n = 3). Data are presented as mean ± SEM. n.s., no significance among all groups.

The concentrations of DA, 5-HT and their metabolites were measured by HPLC to ascertain the effects of NaB and MMF on the striatal neurotransmitters of PD mice. As shown in Figure 4A, MPTP-treated mice had lower concentrations of DA than the control group, and the administration of NaB and MMF stopped the decline in DA levels in PD mice. Likewise, the levels of 5-TH show a similar trend (Figure 4C). In terms of metabolites, the MPTP group had lower levels of DAPOC and 5-HIAA, which were thereafter restored by the administration of NaB and MMF (Figure 4B,D). However, the comparisons shown above only compare trends rather than statistical differences.

### 3.3. NaB and MMF Restored TH Levels and Attenuated Neuroinflammation in PD Mice

**Figure 5:**
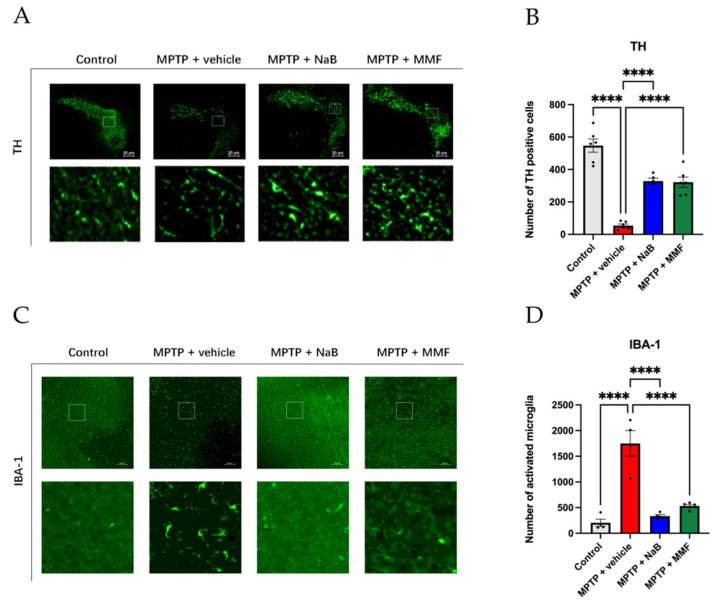
NaB and MMF restore TH levels in PD mice. (**A**) Representative immunofluorescence staining for TH (green). There is a 20 μm scale bar. (**B**) Treatment with NaB and MMF alleviates the reduction of TH+ dopaminergic neurons compared with the MPTP + vehicle group. (n = 5–6). (**C**) Representative immunofluorescence staining for IBA-1 (green) in SNpc. There is a 100 μm scale bar. (**D**) The number of activated microglia was reduced in MPTP + NaB and MPTP + MMF groups compared with the MPTP + vehicle group. (n = 4–5). Data are presented as mean ± SEM. **** *p* < 0.0001 versus the MPTP + vehicle group.

To examine how NaB and MMF influence the survival of dopaminergic neurons, we utilized immunofluorescence labeling to measure the expression of TH in the SNpc. The results of the experiments revealed that, in comparison to the control group, the number of TH-positive dopaminergic neurons in the SNpc in the MPTP + vehicle group considerably decreased (*p* < 0.0001 vs. control) (Figure 5A,B). NaB and MMF therapy, however, relieved this condition (*p* < 0.0001 and *p* < 0.0001 vs. MPTP + vehicle, respectively) (Figure 5A,B). These findings imply that NaB and MMF can prevent animals with PD caused by MPTP from losing TH-positive cells, suggesting their neuroprotective effects.

Neuroinflammation, which is associated with the beginning and development of PD, can be mirrored by microglia activity. Since IBA-1 is a hallmark of active microglia, its level can reflect microglia activity. As shown in Figure 5, the IBA-1 level was higher in the MPTP + vehicle group than in the control group (*p* < 0.0001 vs. control) (Figure 5C,D). In contrast to the MPTP + vehicle group, both NaB and MMF significantly reduced the number of IBA-1 (*p* < 0.0001 and *p* < 0.0001 vs. MPTP + vehicle, respectively) (Figure 5C,D), suggesting lower microglia activity and neuroinflammation after the treatment of NaB or MMF.

### 3.4. Intestinal Barrier Is Destroyed in the PD Mice and Is Alleviated after the Treatment of NaB and MMF

**Figure 6:**
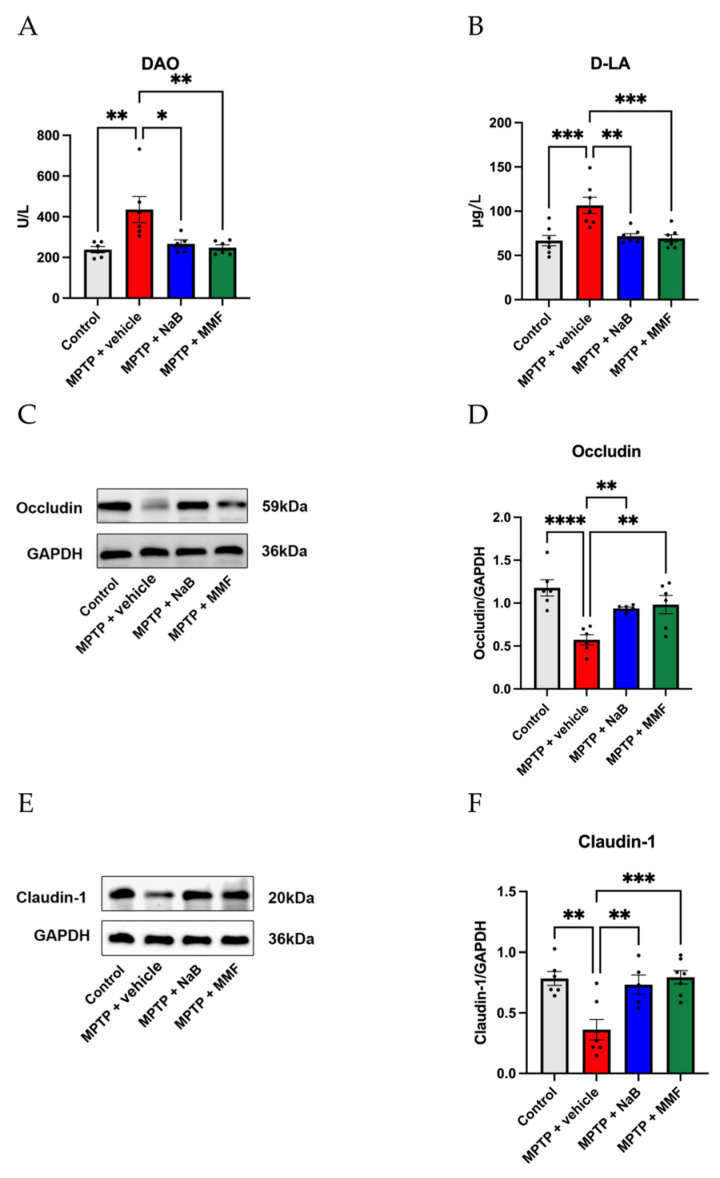
Effects of NaB and MMF on the integrity and permeability of intestinal barrier. (**A**) The content of DAO in each group was detected by ELISA kits. (n = 5–6). (**B**) The content of D-LA in each group detected by ELISA kits. (n = 7). (**C**) Representative immunoblot for Occludin. (**E**) Representative immunoblot for Claudin-1. Alteration of the colon’s intestinal tight junction. Treatment with NaB and MMF increased the expression of the tight junction proteins (**D**) Occludin and (**F**) Claudin-1, which is measured by Western blotting. (n = 5–6). Data are presented as mean ± SEM. * *p* < 0.05, ** *p* < 0.01, *** *p* < 0.001, **** *p* < 0.0001 versus the MPTP + vehicle group.

The intestinal barrier is a wall that blocks harmful substances including bacteria and their metabolites. The tight junction (TJ) is a crucial part of the intestinal barrier. As markers of the integrity of the intestinal barrier, TJ proteins such as occludin and claudin-1 were examined using Western blot analysis. The results illustrated that MPTP-treated animals expressed less occludin (*p* < 0.0001 vs. control) (Figure 6D) and claudin-1 (*p* < 0.01 vs. control) (Figure 6F) than PBS-treated mice. The treatment of NaB and MMF can effectively alleviate the TJ protein loss including occludin (*p* < 0.01 and *p* < 0.01 vs. MPTP + vehicle, respectively) (Figure 6D) and claudin-1 (*p* < 0.01 and *p* < 0.001 vs. MPTP + vehicle, respectively) (Figure 6F). DAO and D-LA were measured as indicators of intestinal integrity. According to the results, as shown in Figure 6, the serum levels of DAO (*p* < 0.01 vs. control) (Figure 6A) and D-LA (*p* < 0.001 vs. control) (Figure 6B) in the MPTP + vehicle group substantially increased when compared to the control group. Compared to the MPTP + vehicle group, the MPTP + NaB group, and the MPTP + MMF group show a remarkably protective impact on the intestinal barrier with decreased levels of DAO (*p* < 0.05 and *p* < 0.01 vs. MPTP + vehicle, respectively) (Figure 6A) and D-LA (*p* < 0.01 and *p* < 0.001 vs. MPTP + vehicle, respectively) (Figure 6B). These results suggest that NaB and MMF gavage can improve intestinal barrier damage in PD mice.

### 3.5. Sodium Butyrate and Monomethyl Fumarate Showed Anti-Inflammation Effects on PD Mice

**Figure 7:**
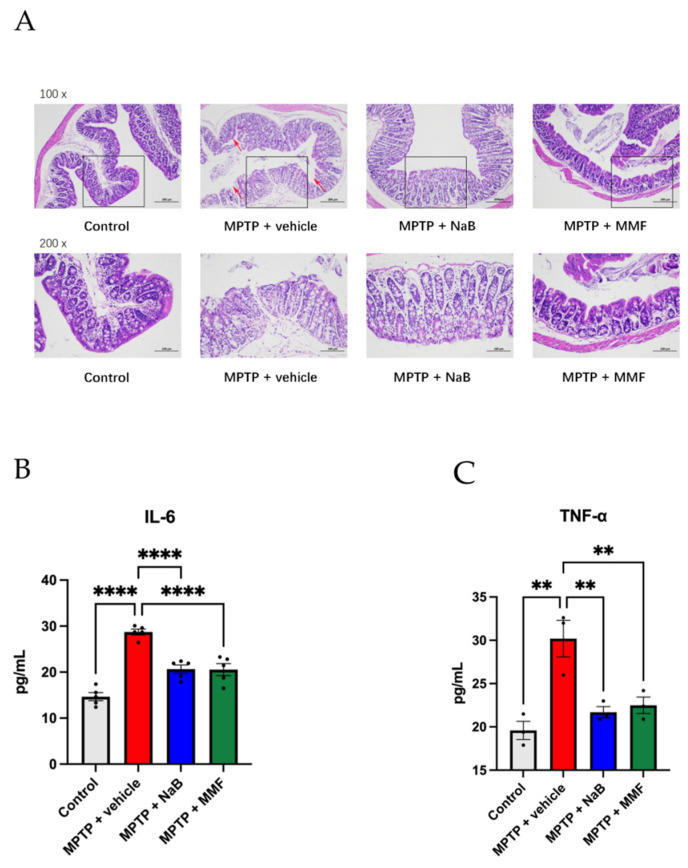
NaB and MMF alleviated colonic inflammation and reduced the level of IL-6 and TNF-α in the serum in PD mice. (**A**) HE staining of colon tissues from different groups, magnifications shown are 100× and 200×; the scale bar represents 100 and 200 μm. (**B**) The levels of IL-6 in the MPTP + NaB group and MPTP + MMF group were significantly lower than those in the MPTP + vehicle group. (n = 5). (**C**) The expression of TNF-α was reduced in the MPTP + NaB group and MPTP + MMF group when compared to the MPTP + vehicle group. (n = 3). Data are presented as mean ± SEM. ** *p* < 0.01, **** *p* < 0.0001 versus the MPTP group.

As shown in Figure 7A, the intact colonic mucosa, regular cell structures, and ordinarily positioned glands in the control group demonstrated the intact and healthy intestinal barrier. However, in the MPTP + vehicle group, the mucosa was harmed and heavily invaded by inflammatory cells with aberrant cell architectures and disrupted glandular organization. The colons of mice given NaB or MMF, however, exhibited minor inflammation and damage.

TNF-α and IL-6 are pro-inflammatory factors in the PD process. Mice treated with MPTP had significantly greater serum levels of TNF-α (*p* < 0.01 vs. control) and IL-6 (*p* < 0.001 vs. control) than mice in the control group. When compared to the MPTP + vehicle group, the NaB and MMF treatment substantially decreased the serum levels of IL-6 (*p* < 0.0001 and *p* < 0.0001 vs. MPTP + vehicle, respectively) (Figure 7B) and TNF-α (*p* < 0.01 and *p* < 0.01 vs. MPTP + vehicle, respectively) (Figure 7C) in MPTP-treated mice, indicating their effects to alleviate systemic inflammation in PD mice.

### 3.6. NaB and MMF Inhibited the NF-κB Pathway Mediated GPR109A to Improve Intestinal Barrier Damage in PD Mice

**Figure 8:**
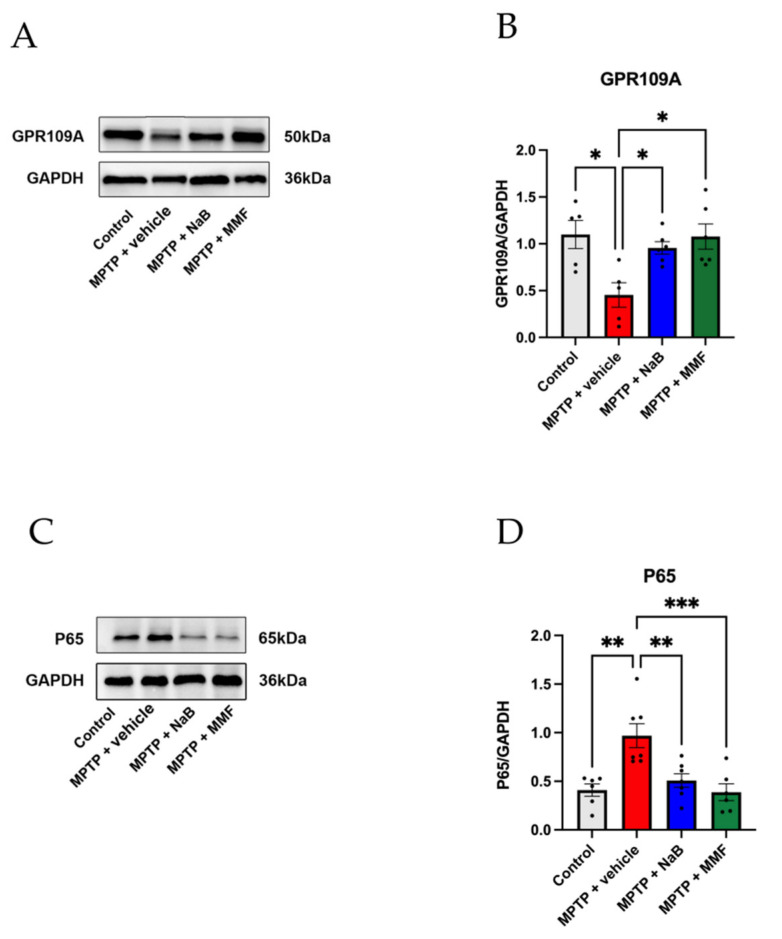
NaB and MMF inhibited the NF-κB pathway-mediated GPR109A to improve intestinal barrier damage in PD mice. (**A**) Representative immunoblot for colonic GPR109A. (**B**) NaB and MMF improve colonic GPR109A expression. (n = 5–6). (**C**) Representative immunoblot for colonic P65. (**D**) NaB and MMF inhibited colonic P65 expression. (n = 6–7). * *p* < 0.05, ** *p* < 0.01, *** *p* < 0.001 versus the MPTP + vehicle group.

The NF-κB pathway is the typical inflammation signaling pathway. When it is activated, MLCK and inflammatory cytokines such as TNF-α are increasingly expressed, which damage the intestinal barrier and increase intestinal permeability [34]. Evidence from the IBD study indicates that NaB interacted with GPR109A to inhibit NF-κB pathway activity. From our perspective, NaB and MMF may share a similar role in PD, namely, they are capable of alleviating the loss of TJ proteins and rebuilding the damaged intestinal barrier in PD. To further demonstrate our opinion, we determined the volume of GPR109A and p65 via Western blot in colon tissue (Figure 8A,C). According to the Western blot, the expression of colonic GPR109A in PD mice was considerably lower than in the control group (*p* < 0.05 vs. control) (Figure 8B). Treatment with NaB or MMF dramatically reversed the loss of GPR109A, indicating that GPR109A was activated in the NaB- and MMF-treated mice (*p* < 0.05 and *p* < 0.05 vs. MPTP + vehicle, respectively) (Figure 8B). At the same time, the activation of GPR109A is related to the inhibition of the NF-κB pathway. The MPTP + vehicle group showed significantly higher expression of p65 than the control group (*p* < 0.01 vs. control) (Figure 8D). However, p65 expression was reduced by NaB and MMF administration (*p* < 0.01 and *p* < 0.001 vs. MPTP + vehicle, respectively) (Figure 8D). These results showed that NaB and MMF are capable of inhibiting the activation of the NF-κB signaling pathway.

## 4. Discussion

**Figure 9:**
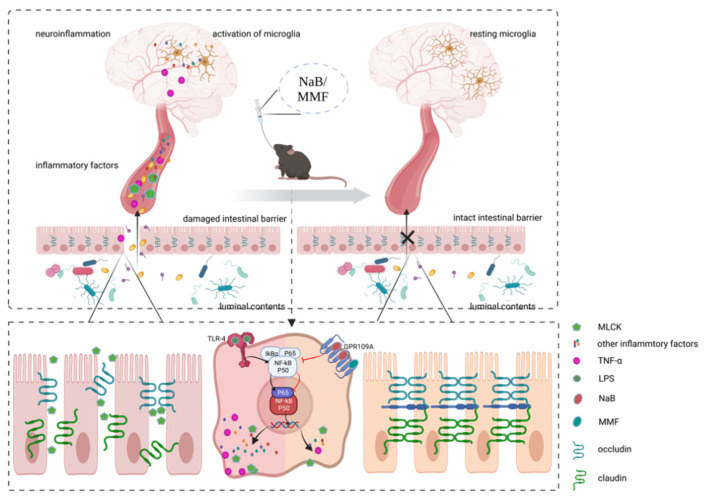
An illustration of the mechanisms. In MPTP-induced PD mice, therapy with NaB or MMF exerted neuroprotective benefits against neuroinflammation and neurodegeneration via stimulating GRP109A and restoring the intestinal barrier. NaB, sodium butyrate; MMF, monomethyl fumarate; MPTP, 1-methyl-4-phenyl-1,2,3,6-tetrahydropyridine; PD, Parkinson’s disease; GPR109A, G-protein-coupled receptor 109A.

Herein, PD model mice were produced through the use of MPTP, and then, the experimental group was treated with NaB for demonstration of its PD-benefit impact and exploration of its exact mechanism. Additionally, in an attempt to investigate the independent positive effect of the GPR109A receptor’s activation, we added a treatment group that used MMF to replace NaB. Our results showed that in MPTP-modeled mice, gavage of NaB or MMF can rescue compromised motor performance and degenerated dopaminergic neurons. The integrity and permeability of the intestinal barrier as well as inflammatory cytokines in blood and glial cells’ activation were also assessed. The intestinal barrier is destructed, and systemic inflammation is exacerbated in PD mice; however, NaB or MMF therapy will ameliorate these effects. It has been suggested that NaB has anti-PD properties. Our findings in this study indicated that gavage of NaB could alleviate motor symptoms and neural pathology with activation of the GPR109A receptor, inhibition of the NF-κB signal pathway, and intestinal barrier repair, suggesting that NaB’s principal mechanisms involve activating GPR109A and then repairing the intestinal barrier. Another intriguing finding is that in PD animals, MMF could also have neuroprotective effects and repairs the damaged intestinal barrier by activating GPR109A, independent of NaB. This confirms the significant role of GPR109A in PD and sheds insight on this receptor that has not drawn widespread attention yet. As shown in Figure 9, we drew a picture to illustrate the mechanisms.

Some pieces of literature have found lower SCFAs and SCFAs-producing bacteria in PD patients, assuming that the supplement of SCFA or the transplanting of healthy bacteria might be therapies for PD [35,36,37]. Butyrate, the most popular SCFA with several important physiological functions, is worth addressing. According to some studies, butyrate may have positive benefits via regulating intestinal dysbiosis and dysfunction [38,39]. Avagliano et al. showed a novel dual-hit model of PD with dysbiosis and gut damage, which is ameliorated after the administration of butyrate [39]. Therefore, butyrate may play a role in Parkinson’s disease. As a form of histone deacetylase inhibitor, sodium butyrate has been shown by Rane et al. to be effective in reducing cognitive deficiencies in pre-motor stage PD [40]. Similarly, Sharma discovered that sodium butyrate can improve cognitive symptoms and motor impairments in PD mice via modulating the histone deacetylase activity [20]. Additionally, as we have depicted above, mitochondrial dysfunction in the brain is prone to result in neurodegeneration. According to certain reports, butyrate can regulate mitochondrial dysfunction and lessen oxidative stress [41,42,43]. Li et al. reported that butyrate can reduce oxidative stress-induced intestinal epithelium barrier injury and mitochondrial damage through the AMPK-mitophagy pathway [42], suggesting a possible mechanism by which NaB could exert its neuroprotective effects via improved mitochondrial function. In these studies, NaB was shown to have positive benefits on PD. In contrast, Qiao discovered that NaB exacerbates PD in PD mice by aggravating neuroinflammation and colonic inflammation [44]. To sum up, there is no consensus on the therapeutic effects of NaB, and more research is still needed to determine how it works exactly. The MPTP + NaB group received gavage treatment of NaB (600 mg/kg/day) after the intraperitoneal injection of MPTP. We found that NaB dosage may have an impact on its effect. In a study published by Hou et al., the effects of giving MPTP mice either a high (2.0 g/kg/day) dose or a low dose (0.2 g/kg/day) of NaB were compared [45]. Hou et al. have found that high doses of NaB had greater positive effects on PD mice’s reduced motor performance and higher levels of TH than low doses of NaB. Therefore, we settled on the strategy of Hu [29]. Our results in this study showed that treatment with NaB or MMF could ameliorate motor symptoms and degenerating dopaminergic neurons in the SNpc or stratum in MPTP-induced PD animals. Immunofluorescence showed that MPTP-induced mice had significantly fewer TH-positive cells in their SNpc, but this decrease was reversed by NaB or MMF, which is consistent with research showing that NaB can prevent the loss of TH-positive cells in PD animals [45,46]. Furthermore, HPLC analysis revealed that the contents of DA, 5-HT and their metabolites in MPTP-induced PD mice will be improved after treatment with NaB or MMF. TH is an important key enzyme in the biosynthesis of dopamine. Due to the severe degradation of dopaminergic neurons, TH-positive cells would decrease in number in PD patients or PD model animals [47,48]. Thus, the expression of TH can mirror both the damage to dopaminergic neurons following MPTP treatment and the therapeutic effects of NaB or MMF. As mentioned above, the substantial death of dopaminergic neurons is one of the distinctive features of PD, causing a deficiency of DA and other neurotransmitters. Neurotransmitters must be in balance to modulate locomotive behavior, while dopamine deficiency will disrupt this balance and bring about the majority of motor symptoms in PD patients [49]. In conclusion, results from immunofluorescence and HPLC show that NaB and MMF can rescue the degeneration of dopaminergic neurons and the decline in TH expression induced by MPTP, suggesting the neuroprotective effects of sodium butyrate and monomethyl fumarate in PD.

The systemic administration of MPTP is the most commonly used method to create a PD model for its effectiveness and accessibility [50,51]. Due to MPTP’s strong liposolubility, it could easily pass the blood–brain barrier [52]. MPP+ is the active form of MPTP, which is likely to bind and damage complex I in mitochondria, which will cause neurotoxicity. In the end, dopaminergic cells in the SNpc will die as a result of an excessively free radical-producing cytotoxic mitochondrial malfunction in glial cells and neurons [53,54]. The majority of clinical symptoms can therefore be mimicked by MPTP, particularly motor dysfunctional symptoms such as tremors and bradykinesia [55]. Acute, chronic, and subacute regimens are the three types of regimens avail for the creation of PD models by MPTP [27,48,56,57]. The subacute regimen is the most popular of these three model techniques due to its security and effectiveness. The doses of 20 mg/kg/day and 30 mg/kg/day are both widely used for subacute regimens. Our preliminary experiments show that our C57BL/6J mice are prone to a high percentage of mortality at the MPTP dose of 30 mg/kg/day. Therefore, we chose a dose of 20 mg/kg/day for ethical reasons and the rational use of experimental materials. In the current study, three different kinds of behavior trials were used to appraise the motor symptoms of MPTP-modeled mice. Separately, shorter latency time in the rotarod test reflects fatigue, longer time in the pole test reflects bradykinesia, and a lower score in the traction trails mirrors tremor. Taken together, behavior results show the substantial locomotor abnormalities caused by MPTP injection, which can be ameliorated obviously by the use of NaB or MMF.

The pivotal role of the intestinal barrier in PD pathogenesis has drawn increasing attention recently [58,59]. However, papers that treat PD that are based on the repair of the intestinal barrier are rare. In accordance with previous studies discovering the damage to the intestinal barrier in PD patients or PD animals [17,60], we observed decreased TJ protein expression in the distal colon, as well as increased content of diamine oxidase (DAO) and d-lactate (D-LA) in serum after MPTP administration, implying the dysfunction of the intestinal barrier in PD mice. The benefits of NaB or MMF to protect and rebuild the intestinal barrier are demonstrated by the reversal of decreased colonic TJ proteins and elevated serum DAO or D-LA following administration of the substance. Covering approximately 400 m^2^, the intestinal barrier is vital to the homeostasis of our human body as the largest barrier separating the lumen content from the internal environment [61]. The barrier has bifarious functions including absorption and limitation: nutrients, water, and electrolytes digested from food or drink could be able to cross the barrier through transcellular transport easily and without restraint [62]. Furthermore, the barrier is exposed to large amounts of bacteria, harmful bacterial byproducts such as endotoxin (LPS), and other foreign bodies that enable immunoreaction or inflammation [63]. Therefore, blocking these unnecessary substances from entering the internal environment is another crucial function of the intestinal barrier. Given the destroyed epithelial cells and the cleavage of intercellular junction proteins, abnormally profuse unfavorable contents in the gut lumen will penetrate into the underlying tissue and elicit an inflammatory response [64]. It is also interesting that a number of pathologic diseases are linked to intestinal barrier dysfunction. For instance, a number of illnesses, including IBDs, asthma, allergic rhinitis, and Alzheimer’s disease, have been linked to a compromised intestinal barrier [65]. As we have already listed, many diseases are influenced by compromised intestinal barrier, and PD is not an exception. Recent studies have documented the compromised intestinal barrier in PD patients or PD animals [15,17,21]. Moreover, Hasegawa et al. reported lowered LPS binding protein (LBP) in PD patients [66], indicating long-term enhanced permeability in PD patients’ intestinal barrier. These results increased the likelihood that the gut barrier and Parkinson’s disease are related. An essential element of the intestinal barrier is the TJ, which controls the passage of molecules and other tiny particles between adjacent epithelial cells [25]. Occludin, as well as claudin, are two major TJ proteins that keep the gut barrier function via sealing the intercellular space between neighboring epithelial cells [61]. Occludin, an adhesive transmembrane protein, is often used as a TJ marker protein and appears to be a substantial component of all TJs. Although its physiological role in TJ is unknown, some scholars hold the view that occludin might be involved in TJ assembly [67]. Claudins, which make up TJ strands, are essential for controlling paracellular permeability. Additionally, the cytoplasmic portion of claudins interacts with the zonula occludens family of scaffolding proteins [68]. Intact TJ structure is necessary for the maintenance of barrier function; otherwise, bacteria may translocate due to TJ degradation. DAO is frequently employed as a circulating marker for the permeability of the intestinal barrier since it is an enzyme that is abundant in the gastrointestinal mucosa of humans and other mammals [69]. D-LA is a type of metabolic byproduct produced by gut flora via bacterial glycolysis [70]. When the permeability of the gut barrier is severely elevated, DAO and D-LA in the lumen easily cross the intestinal barrier and enter the bloodstream [71,72]. The results of the ELISA test showed that the blood of PD mice had higher levels of DAO and D-LA, which significantly decreased after being treated with NaB or MMF. In a word, TJ protein expression in gut tissues represents the integrity of the intestinal barrier, whereas blood levels of DAO and D-LA show intestinal barrier permeability. Our findings suggest that intestinal barrier failure in mice treated with MPTP may contribute to the degeneration of SNpc neurons and function as a risk factor for Parkinson’s disease. Following MPTP injection, TJ proteins in the colon, such as occludin and claudin-1, decreased while blood levels of DAO and D-LA dramatically rose, reflecting the destruction of the intestinal barrier in MPTP mice. These findings specifically demonstrated that the intestinal barrier’s integrity had been compromised and that its permeability had increased. As illustrated above, an intestinal barrier that is sound and intact is the foundation for the homeostasis of host tissue. A vicious cycle will be formed because of barrier dysfunction. When the intestinal barrier is damaged, its permeability will be increased and luminal contents will passage the intestinal barrier easier. Inflammatory processes are intrigued because the intestinal luminal contents that are normally separated from subepithelial tissue translocate into the deeper layer. Finally, because subepithelial inflammation contributes to maintaining both broken and open barriers, a vicious cycle is established [73]. However, after receiving NaB or MMF treatment, the compromised intestinal barrier in MPTP-induced mice would be reconstructed. According to our research, the advantages of NaB or MMF on the intestinal barrier may be due to the activation of GPR109A and suppression of the NF-κB signaling pathway, as is illustrated later. 

Additionally, when the intestinal barrier is destroyed, it is terrible that inflammation will not limit itself in the gut; instead, it will gradually invade the nervous system via systemic inflammation. Inflammatory markers such as TNF-α are more prevalent in PD patients, indicating a chronic inflammatory process [74]. A noticeable pathological change in the PD brain is the aberrant activation of microglia, which indicates neuroinflammation in the brain. It is possible that the abnormal activation of glia cells is a key link connecting local intestinal inflammation and central nervous system (CNS) inflammation. Therefore, the degree of glia cell activation can both represent the inflammation status of the CNS and reflect the dysfunction of the intestinal barrier. As a marker of activation in microglia, IBA-1 can be used to detect the number of active microglia [75]. Results showed higher IBA-1 in MPTP-induced mice, which was reduced by NaB or MMF therapy.

It is clear from the literature and our research that intestinal barrier failure and secondary vicious cycles play paramount roles in PD. Thus, a promising direction for PD therapy would be to treat the compromised barrier. In the current research, we attempted to protect and rebuild the dysfunctional intestinal barrier by activating GPR109A, finally breaking the downward spiral, which is depicted below, and reducing neurodegeneration. MMF is the bioactive metabolite of dimethyl fumarate (DMF). It is intriguing that DMF is approved by the FDA to treat multiple sclerosis, another neurodegenerative disease [76]. Ahuja et al. reported that MMF can block dopaminergic neurodegeneration in MPTP-induced animals via the Nrf2 signaling pathway, raising the prospect that it could be used as a novel PD treatment [77]. According to our conjecture, the activation of GPR109A may be a key additional mechanism via which MMF exerts its anti-PD effects. Our study found decreased colonic GPR109A expression and increased colonic p65 expression in PD mice, which suggests that the NF-κB signal pathway is more activated and that GPR109A is less activated. Nevertheless, after the treatment of NaB or MMF, colonic GPR109A expression is obviously elevated and NF-κB activation is suppressed. As one of the most common ligands for the GPR109A receptor, niacin is considered to be helpful in PD. Giri et al. reported niacin’s anti-PD effect via GPR109A’s regulation of inflammation [78]. Additionally, they found that PD patients’ white blood cells had GPR109A upregulation, a sign of systemic inflammation [79]. Additionally, Wakade et al. reported that low-dose niacin supplementation is capable of managing PD symptoms via regulating GPR109A [80]. However, no research has connected the repair of the intestinal barrier to the possible therapeutic effects of GPR109A in PD. Various studies have been conducted in order to ascertain NaB’s anti-PD effect and to clarify its underlying mechanisms. For instance, it has been noted in several studies that NaB exerts beneficial effects as a histone deacetylase inhibitor [81,82]. We first demonstrated that NaB’s anti-PD effect is caused by the activation of GPR109A and the repair of the intestinal barrier. NaB is another typical GPR109A ligand that has been demonstrated to preserve gut barrier integrity and reduce local inflammation in the gut [24]. According to several studies, using NaB will promote TJ protein expression with or without GPR109A activation [22,25,83,84]. Therefore, by protecting and rebuilding the intestinal barrier through the activation of GPR109A, NaB may be able to break the vicious cycle induced by gut barrier malfunction and stop the pathological progression of PD. We first found the decreased expression of colonic GPR109A in MPTP-induced mice, reflecting the under-activation of GPR109A. This decrease was then reversed by giving NaB or MMF, and the PD mice’s damaged intestinal barriers were restored, demonstrating the critical role of GPR109A in PD and intestinal barrier restoration.

## 5. Conclusions

Taken together, our study showed that a compromised intestinal barrier is involved in the pathogenesis of PD through a leaky gut, which will incur local and systemic inflammation. Furthermore, we show that by lowering the NF-κB signaling pathway’s activity, activation of GPR109A by NaB or MMF can reverse the destruction of the intestinal barrier and ameliorate PD symptoms. In the current study, we showed how repairing a damaged intestinal barrier can be a promising PD treatment strategy. Second, our research identified the mechanism by which NaB exerts its anti-PD actions, which depends on GPR109A receptor activation. Namely, the current study specifically identifies the precise protective role of GPR109A in PD, offering a novel method for the treatment of PD. More research that focuses on PD’s defective intestinal barrier and its treatment by stimulating the GPR109A receptor is nevertheless required.
